# A comparative analysis of microplastic contamination in hermit crab *Clibanarius rhabdodactylus* Forest, 1953, inhabiting intertidal and subtidal Coastal habitat of Gujarat state

**DOI:** 10.1371/journal.pone.0325324

**Published:** 2025-06-12

**Authors:** Jaimin Parmar, Krupal Patel, Vasantkumar Rabari, Ashish Patel, Dipak Kumar Sahoo, Jigneshkumar N. Trivedi

**Affiliations:** 1 Department of Life Sciences, Hemchandracharya North Gujarat University, Patan, Gujarat, India; 2 Marine Biodiversity and Ecology Laboratory, Department of Zoology, The Maharaja Sayajirao University of Baroda, Vadodara, Gujarat, India; 3 Department of Veterinary Clinical Sciences, College of Veterinary Medicine, Iowa State University, Ames, Iowa, United States of America; University of Basrah, IRAQ

## Abstract

Microplastics (MPs) contamination has increased significantly due to inadequate plastic waste, leading to MPs infiltration in marine organisms. Crustaceans, especially the anomuran crabs, represented significant benthic communities in the intertidal zone. The current investigation aims to check the variation in MP contamination in Hermit crab *Clibanarius rhabdodactylus* inhabiting the intertidal and subtidal zone of Gujarat state, India. A total of 50 crabs (25 males and 25 females) of *C. rhabdodactylus* were collected from both zones along the coast of Gujarat in January and February 2024. In the laboratory, hermit crabs were weighed, dissected, and processed for tissue digestion. Sediment and water samples also underwent digestion. All samples were then processed through flotation, filtration, microscopic observation, and chemical characterization. The higher contamination was recorded in intertidal specimens than in subtidal specimens. The MP contamination in *C. rhabdodactylus* varied significantly between intertidal and subtidal specimens. MP contamination in both habitats was greater in females than in males. MP contamination in sediment and water was higher in the intertidal region than in the subtidal region, with no significant difference. Morphometric examination of MPs indicated the maximum abundance of fibers in terms of MP shape, followed by fragments and films. Black coloured MPs with 0.5–1 mm size were recorded dominantly in both habitats. The isolated MPs were primarily composed of polyethylene and polypropylene polymers. This study proposed immediate measures to address the issue of effective management of plastic litter in the marine ecosystem of the state. The present study revealed that MPs are widely distributed in the intertidal region and possess a greater risk of MP accumulation than those in the subtidal region.

## 1. Introduction

The extensive range of applications and insufficient management of plastic byproducts have led to widespread dispersion of plastic waste in both land-based and oceanic environments [1]. As reported by Plastic [2], the current rate of plastic production exceeds 400 million metric tons annually. Microplastics (MPs) are plastic particles ranging from 0.1 µm to 5 mm in size [3]. According to their source, MPs are further divided into primary and secondary classes [4]. Primary MPs are purposefully manufactured for specific applications [5]. In contrast, large plastic degradation and breakdown lead to the formation of secondary MPs [6]. MPs display various shapes and forms, such as fibers, films, forms, fragments, and granules [7]

Microplastics (MPs) are widespread in various environments, including coastal regions. Beach sediments contamination with MPs raises a significant environmental issue in India. Anthropogenic activities have contributed to the presence of MPs in beach sediments on various Indian coasts, showing that more tourist beaches have more contamination than less disturbed coastal areas along the coast [8–10]. Evidence of MP pollution has been found in various organisms, including crabs, shrimps, prawns, fish, mollusks, and bivalves [11–20]. MPs can cause harmful physical effects in animals, such as oxidative stress, reduced growth and development, physical injuries, loss of appetite, cellular damage, and intestinal obstruction [21].

Researchers have discovered that MPs can significantly affect the behaviour of hermit crabs [22]. Acute exposure to MPs may deteriorate the ability to select the right shells, disrupt the decision-making processes, and affect survival behavior [22]. The exposure of MPs shows various behavioral changes in hermit crabs, including predator avoiding, shell selection cues, rapping strength, and other factors [23,24]. These findings highlight the widespread and relevant effects of MP pollution on hermit crabs, showing the urgent need for further research and conservation initiatives to mitigate these effects.

Gujarat is situated on the westernmost coast of India and is bordered by the Arabian Sea. Its coastline spans approximately 1650 km, making it the state with the longest coastline in India [25]. Gujarat relies on its coastal regions for fishing, salt production, and shipbuilding. The Somnath Temple, Diu, and Dwarka beaches attract tourists worldwide; hence, the coastal community actively engages in the tourism sector [26]. The state offers a variety of coastal environments, including rocky and muddy intertidal regions, mangrove coral reefs, and sandy beaches. The environments are home to a wide range of intertidal organisms [27]. The coastal region includes the intertidal zone (from high tide and low tide marks) and subtidal zone (which stretches from the tide mark to the continental shelf at a depth of approximately 200 m). The coastal regions of Gujarat revealed that the majority of beaches are contaminated by MPs [16,28]. Additionally, researchers have found varying levels of contamination in major biota, such as *Harpadon nehereus* [29], *Portunus sanguinolentus* [30], *Saccostrea cuccullata* [19], and various prawn species [31]. A total of 115 hermit crab species have been recorded in India [27]. Hermit crabs play a significant role in their ecosystem, including nutrient cycling, habitat maintenance, and prey-predator dynamics, and serve as bioindicators [27,32,33]. Based on a literature review, a single study has been conducted to assess the MP contamination in hermit crabs found in Sundarban Biosphere Reserve, India, highlighting the need for further investigation on MP contamination in different species of hermit crabs in India [34].

The hermit crab species *Clibanarius rhabdodactylus* Forest, 1953, is the most common species in the coastal area of Gujarat [27]. It is distributed from Jakhau (Kachchh) to Nagao beach (Diu), which occupies both the intertidal and subtidal habitats [35]. *C. rhabdodactylus* contains a varied diet that includes detritus, algae, and small invertebrates, indicating that the species exhibits a generalized feeding habit [33]. This dietary behaviour increases to ingest the MPs along with detritus, resulting in the accumulation of MPs in its body. Therefore, it is crucial to understand the level of MP’s contamination in the *C. rhabdodactylus* population in intertidal and subtidal habitats along the Gujarat coast.

## 2. Materials and methods

### 2.1 Study area

**Fig 1:**
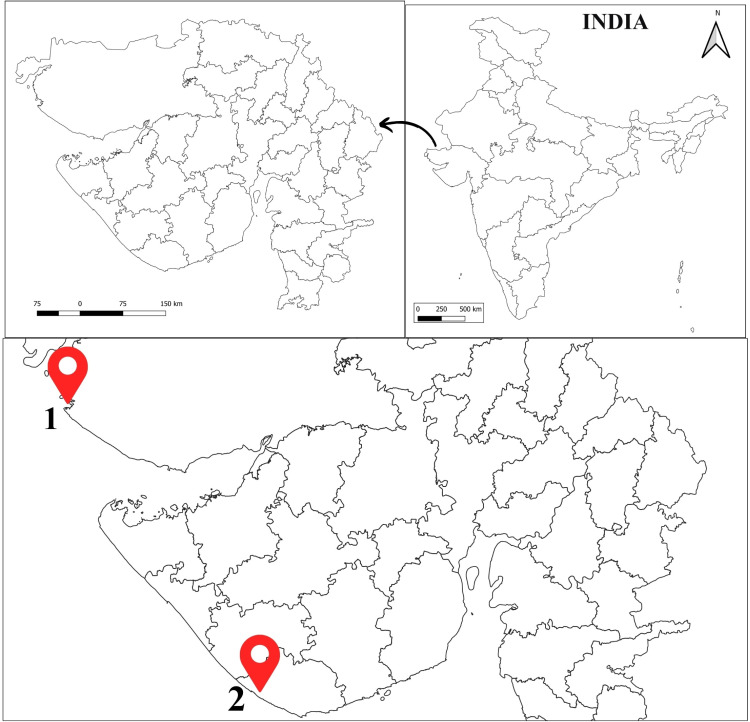
Map of the study sites: 1. Jakhau, 2. Veraval along the coast of Gujarat, India.

*C. rhabdodactylus* is abundantly found in the region of Veraval (20°54’36.0”N, 70°21’07.7”E) in Gir Somnath district, the state’s biggest fishing port, and the subtidal region of Jakhau (23°13’58” N, 68°36’42” E) of Kachchh district, one of the oldest ports, which plays a crucial role in sustaining the local community’s life through fishing activities (Fig 1).

### 2.2 Sampling methods

During low tide, crabs were randomly sampled from the intertidal region using the handpicking method. No permits were required to conduct this work, as the studied species is not protected under the Wildlife Protection Act of India, 1972. With the assistance of fishermen, specimens from the subtidal region (25–100 m depth) were collected using fishing boats and trawl nets. A total of 50 crabs (25 males and 25 females) of *C. rhabdodactylus* were collected from both zones along the coast of Gujarat in January and February 2024. Five sediment samples were collected at each sampling station with 100 meters distance from one sampling point to another. A stainless-steel sampler packed it to the top. To ensure consistency, a stainless-steel sheath was inserted under the sampler at each collection time. A total of 15 L of surface water samples were collected from each site in triplicate. The collected surface water was sieved through a 25 µm mesh, filtered, and transferred to a glass container. The collected specimens were transported to the laboratory in an ice box for subsequent analysis.

### 2.3 Laboratory analysis

#### 2.3.1 MP extraction, identification, and quantification.

**Fig 2:**
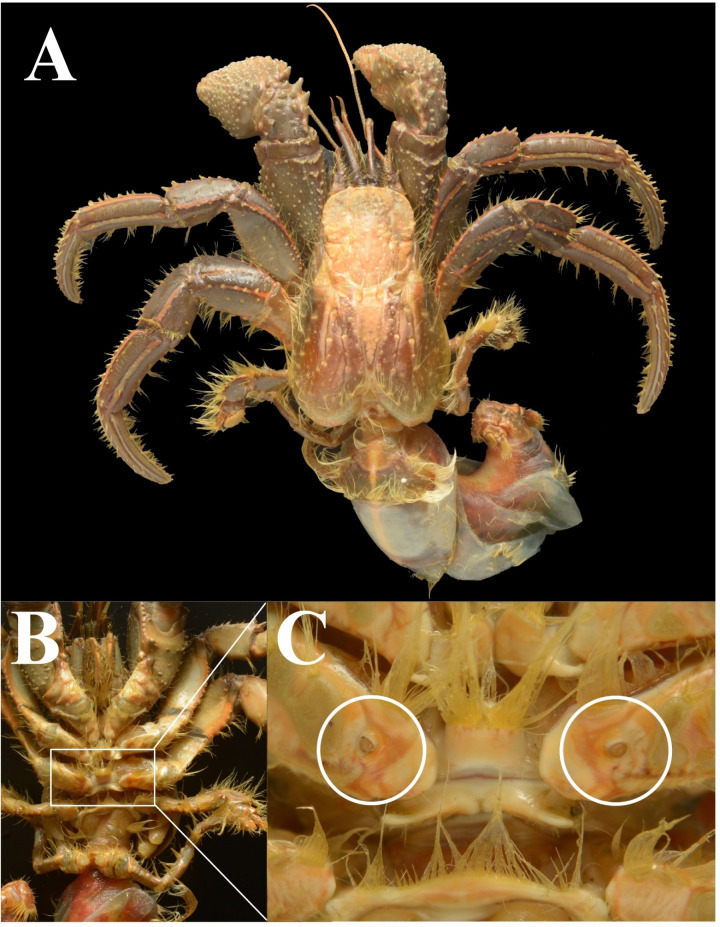
*Clibanarius rhabdodactylus* Forest, 1953: A. dorsal view of male, B. ventral view of female, C. gonopores on coxa of 2^nd^ pair walking legs.

Once the specimens were brought to the laboratory, hermit crabs were removed from their shells for further analysis (Fig 2A). After removal from the shells, the species was identified based on available standard taxonomic keys [36]. Furthermore, the sex of the crabs was identified and categorized into males (absence of gonopore on the coxa of the 2^nd^ pair of walking legs) and females (presence of gonopore on the coxa of the 2^nd^ pair of walking legs) (Fig 2B, C). A total of 50 crabs (25 males and 25 females) of *C. rhabdodactylus* from the intertidal and subtidal regions were analyzed to assess the differences in MP contamination from different coastal regions. Digital Vernier calipers were used to record the shield length of each specimen as a measure of body size. Each specimen was dissected to remove its appendages (chelipeds and walking legs), and the weight was measured using a digital weight balance. Ten sets (Five crabs each set) were prepared separately for males and females collected from the intertidal and subtidal habitats.

Each sample of sediment, water, and whole body of hermit crab was treated with a 10% potassium hydroxide (KOH) solution and digested at 60 °C for 48 hours in a hot air oven [37]. The digested tissue samples were then floated with a supersaturated sodium chloride (NaCl) solution. Microplastics (MPs) were allowed to float for 24 hours according to the density gradient. The supernatant was then filtered through Whatman filter paper (pore size: 20 µm). After filtration, the filter papers were placed in a petri dish and allowed to dry at room temperature. After drying, the filter papers were examined under a stereomicroscope for the morphological characterization of MPs, which includes their shape, size, and color. The size of MPs was measured using an ocular micrometer attached to a stereomicroscope.

The polymer composition of recovered MPs was identified using ATR-FTIR (Bruker-Alpha). 10% of the recovered MP samples from each representative class were analyzed [28]. The obtained spectra were compared with a library of known primary and secondary plastic polymers (FLOPP AND FLOPP-e; n = 762 spectra) [17]. A spectral match greater than 70% was considered indicative of MPs [38].

### 2.4 Contamination control

Necessary precautions were strictly followed to avoid possible contamination during sample collection and laboratory analyses. All specimens were cleaned thoroughly with distilled water to remove any unwanted objects from the sample surface in subsequent steps. Throughout the process, metal trays, stainless steel, and glass instruments were utilized and cleaned adequately with Milli-Q water before use. To minimize environmental pollution, the laboratory study was conducted in a confined facility with minimal human activity [30]. The person conducting the laboratory analysis wore nitrile gloves and a white cotton apron to prevent MP contamination from the synthetic clothes. The hot needle method was used to confirm the extracted MPs. Three blanks were placed at each step to assess contamination in the air. If MPs were recovered from the blanks, their mean was calculated and subtracted from the total number of MPs recorded on each filter paper. However, it was found that there was no presence of any MPs in the blank samples.

### 2.5 Data analysis

The concentration in terms of items/g was calculated to evaluate the abundance and standard error of MPs in different environments and between sexes within each habitat. The Shapiro-Wilk test was used to assess the normality of the obtained data. The results indicated that the data were not normally distributed (p < 0.05); therefore, non-parametric tests were used. A Mann-Whitney (U) test was performed to evaluate differences in MP contamination between the coastal habitats, while a Kruskal-Wallis test was conducted to investigate the variation in contamination between the sexes within each habitat. The percentage composition of MPs’ size, shape, and color was determined to compare MPs across sexes and coastal habitats. All statistical analysis was conducted using Microsoft Excel and PAST software (version 4.03).

#### Pollution indices.

Contamination Factor (CF), Polymeric Risk Assessment or Hazardous Index (H), and Pollution Risk Index (PRI) were utilized to evaluate the extent of MP pollution between intertidal and subtidal regions (S1 Table).

#### Contamination Factor (CF).


CFI = Ci/Co (1)

Here, *i* represents the study site, *Ci* indicates the abundance of MPs at each site, and *Co* denotes the minimum MPs abundance observed. Due to the unavailability of prior baseline data on MPs concentrations in the same environment, the lowest MPs abundance recorded during this study was used as the *Co* value for calculations.

#### Polymeric Risk Assessment or Hazardous Index (H) and Pollution Risk Index (PRI).

Hazardous scores were calculated to evaluate the polymeric risks and toxicity levels of MPs in the environment. This assessment was based on the concentration of MPs and their chemical composition.


$$\begin{array}{c} H_{i}\;=\;\Sigma \;(p_{ji}/Ci)\;x\;S_{j} \end{array}$$ (2)

In this context, *pji* represents the number of MP polymers identified at each study site, while the coefficient *Sj* corresponds to the risk score assigned to each specific plastic polymer identified. The risk scores for the identified MP polymers are as follows: polyethylene = 11, polypropylene = 1, and ethylene-vinyl acetate = 9.


$$\begin{array}{c} PRI_{i}\;=\;H_{i}\;\times \;CF_{i} \end{array}$$ (3)

## 3. Results

MP contamination in *C. rhabdodactylus* from two different coastal regions, the intertidal and subtidal regions, was estimated in the present study. The average size (shield length) of *C. rhabdodactylus* was 3.48 ± 0.09 mm for intertidal specimens and 7.81 ± 0.16 mm for subtidal specimens. The average size of the males was significantly larger than that of the females in the intertidal and subtidal regions (Mann-Whitney U = 29; z = 8.41; p < 0.001) (Mann-Whitney U = 9.21; p < 0.01) (Table 1).

**Table 1:** Abundance of microplastic contamination in *Clibanarius rhabdodactylus* samples collected from two different regions of the coastal habitat of Gujarat state, India. (**p < 0.01).

Parameters	Intertidal		Subtidal	
	Male	Female	Male	Female
**Number of individuals**	25	25	25	25
**Total MPs**	323	321	187	288
**Mean Shield Length (mm)**	4.24 ± 0.08**	2.72 ± 0.06**	8.35 ± 0.14**	7.26 ± 0.23**
**Mean weight (g)**	3.65** **± 0.84	2.54 ± 0.41	7.89 ± 2.06	6.98 ± 1.47
**items/g**	9.21 ± 1.21**	13.10 ± 1.58**	2.40 ± 0.26**	4.25 ± 0.48**
**items/Individual**	6.46	6.42	3.74	5.76
**Grand Total**	644		475	

### 3.1 MPs abundance

Among the examined crabs of *C. rhabdodactylus,* the maximum number of MPs was recorded in the samples collected from the intertidal region compared to the subtidal region. Moreover, the total number of MPs recorded from females was higher than that from males (Table 1).

**Fig 3:**
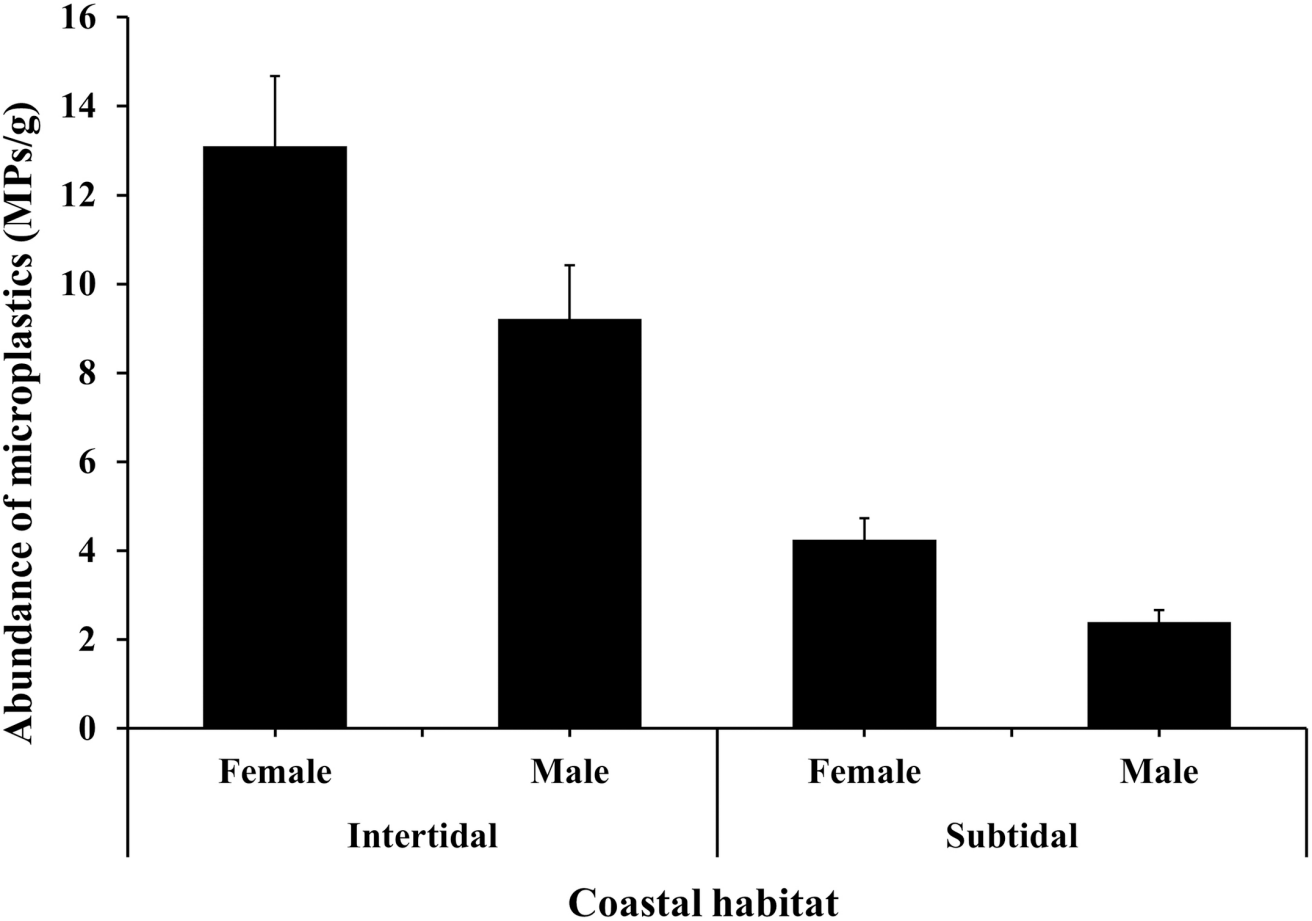
Abundance of microplastics found in *Clibanarius rhabdodactylus* samples collected from different coastal habitats of Gujarat State, India.

**Fig 4:**
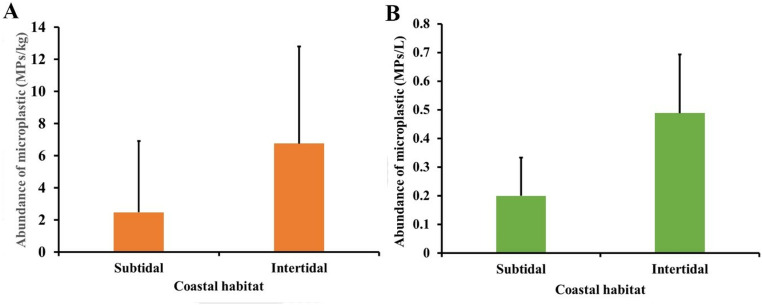
Abundance of microplastics in intertidal and subtidal regions: A) sediments and B) water.

The mean abundance of MP contamination in the *C. rhabdodactylus* crabs collected from the intertidal region (11.16 ± 1.06 items/g) was significantly higher compared to the subtidal region (3.33 ± 0.34 items/g) (Mann-Whitney U: 92.5; p < 0.01). The contamination rate of MPs varied significantly among the sexes of both region with maximum MP abundance observed in the female crabs (13.1 ± 1.57 items/g) collected from the intertidal region, followed by the males (9.21 ± 3.83 items/g) of the intertidal region, females (4.25 ± 0.45 items/g) of the subtidal region, and males (2.39 ± 0.25 items/g) of subtidal region (Kruskal-Walis, H(χ2): 14.69; p < 0.01) (Fig 3). The average abundance of MP contamination in sediment was recorded as higher in intertidal (6.75 ± 6.04 items/kg) than subtidal (2.47 ± 4.44 items/kg) (Fig 4A). The abundance of MP contamination in sediment did not vary significantly between the intertidal and subtidal areas (Mann-Whitney U: 4; p = 0.08). The average abundance of MP contamination in water was recorded as higher in intertidal (0.48 ± 0.20 items/L) than subtidal (0.2 ± 0.13 items/L) (Fig 4B). The abundance of MP contamination in water did not vary significantly between the intertidal and subtidal areas (Mann-Whitney U: 1; p = 0.2).

#### Pollution indices.

The pollution indices were calculated to understand the level of contamination and toxicity of polymers between intertidal and subtidal samples. The CF revealed the considerable (CF = 3.35) and moderate contamination (CF = 1) in intertidal and subtidal regions, respectively. Findings of the H index revealed that both regions were in risk category III. While, based on the findings of PRI, intertidal and subtidal regions fell under very high and medium risk categories, respectively.

### 3.2 Physical characterization of MPs

#### 3.2.1 Shape of MPs.

**Fig 5:**
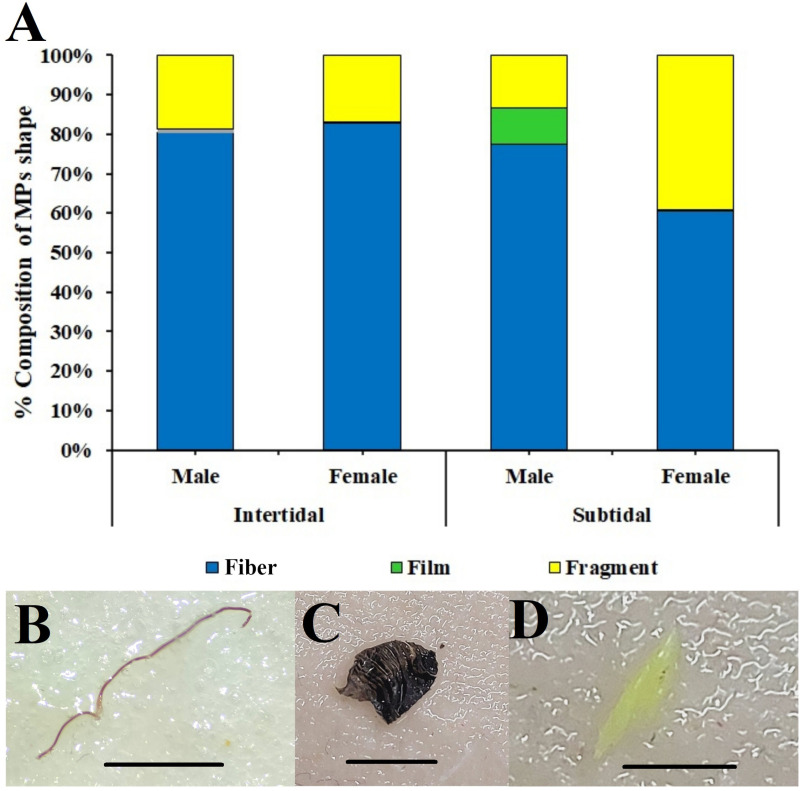
Shape of microplastics found in *C. rhabdodactylus* samples collected from different coastal habitats of Gujarat state, India. A) Percentage composition of shapes of MPs, B) fibers, C) films, and D) fragments. (Scale bar = 1 mm).

**Fig 6:**
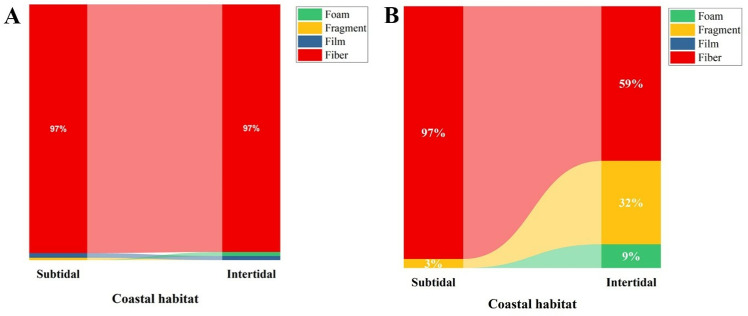
Percentage composition of microplastic shapes: A) sediment and B) water.

Shape-wise quantification revealed three different forms of MPs: fibers, fragments, and films. Among these, fibers (75.48%) were the most abundant MPs in both regions, followed by fragments (22.08%) and films (2.44%) (Fig 5). Sex-wise comparison of *C. rhabdodactylus* in the intertidal region revealed that fibers (81.11%) were the most abundant type of MPs in males, followed by fragments (18.89%), while in females, fibers (82.86%) were the most abundant, followed by fragments (0.32%) and film (16.82%). Similarly, in the case of *C. rhabdodactylus* in the subtidal region, fibers (77.54% and 60.42%) were the highest, followed by fragments (9.09% and 0.35%) and films (13.37% and 39.24%) in males and females, respectively (Fig 5). Similarly, fibers were found predominantly in the sediment and water samples collected from the intertidal and subtidal region (Fig 6A and B).

#### 3.2.2 Size of MPs.

**Fig 7:**
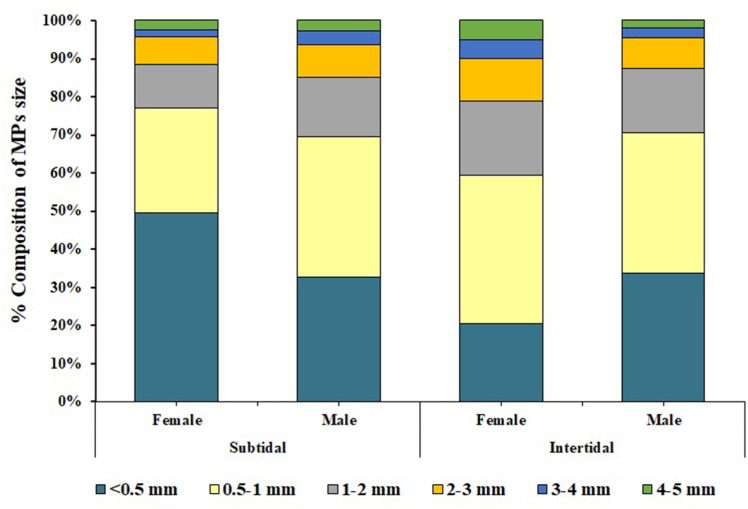
Size of microplastics found in *Clibanarius rhabdodactylus* samples collected from different coastal habitats in Gujarat state, India.

**Fig 8:**
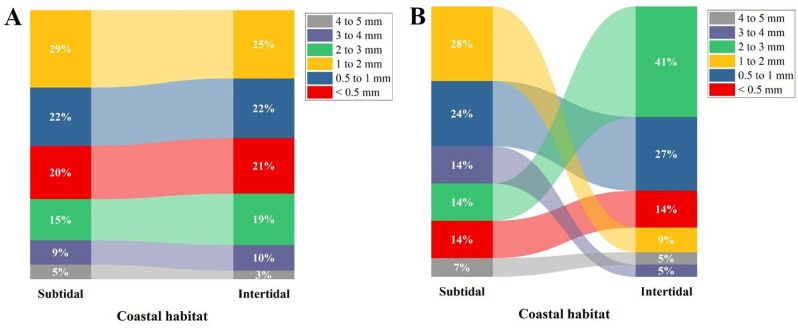
Percentage composition of microplastic size: A) sediment, B) water.

MPs classification based on size showed that the intertidal region exhibited the maximum amount of MPs ingestion in the 0.5–1 mm size class, followed by <0.5 mm, 1–2 mm, 2–3 mm, 3–4 mm, and 4–5 mm size classes. Similarly, in the crabs *C. rhabdodactylus* collected from the subtidal region, the maximum number of MPs belonged to the < 0.5mm size class, followed by the 0.5–1 mm, 1–2, 2–3, 3–4, and 4–5 mm size classes (Fig 7). In the case of size-wise classification of MPs in sediment and water, various sizes MPs were found in the intertidal and subtidal regions (Fig 8A and B).

Assessment of MP contamination in different sexes of *C. rhabdodactylus* crabs collected from the intertidal region showed that maximum MPs in males and female crabs were of 0.,5–1 mm size class, followed by <0.5 mm, 1–2 mm, 2–3 mm, 3–4 mm, and 4–5 mm size classes. In the subtidal region, the maximum MPs recorded in male crabs were of the 0.5–1 mm size class, followed by the < 0.5, 1–2, 2–3, 3–4, and 4–5 mm size classes. On the other hand, maximum MPs recorded in female crabs were of <0.5 mm size class, followed by 0.5–1 mm, 1–2 mm, 2–3 mm, 3–4 mm, and 4–5 mm size classes (Fig 7).

#### 3.2.3 Colour of MPs.

**Fig 9:**
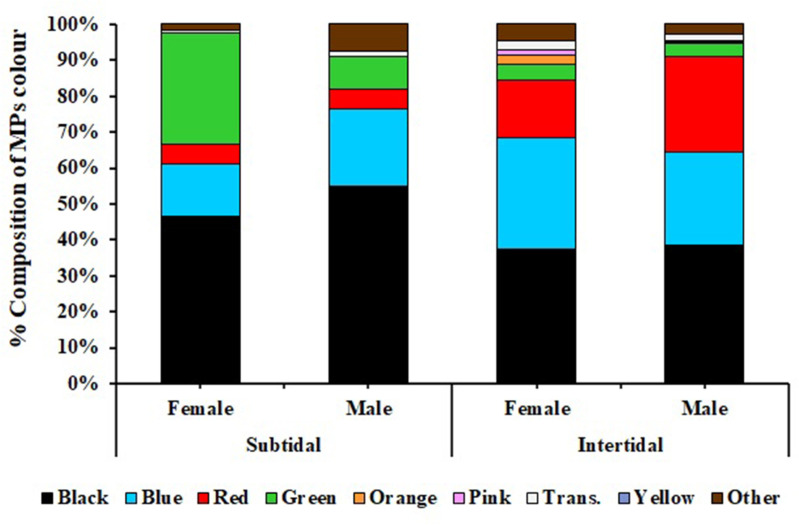
Color of microplastics found in *Clibanarius rhabdodactylus* samples collected from different coastal habitats in Gujarat State, India.

**Fig 10:**
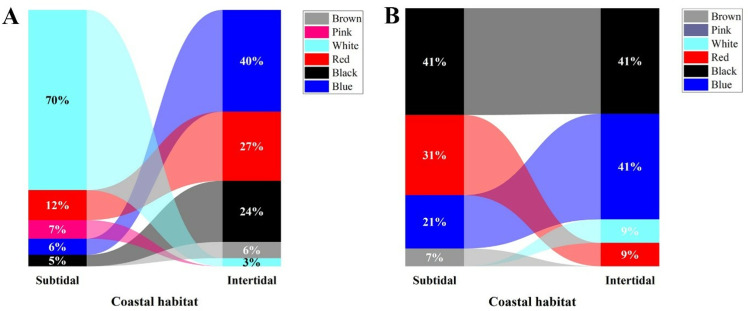
Percentage composition of color of microplastic: A) sediment, B) water.

Based on colour, black MPs predominantly appeared in the intertidal zone, followed by blue, red, green, transparent, and others. In contrast, the MPs obtained from *C. rhabdodactylus* crabs collected from the subtidal region revealed that black MPs were dominant in the subtidal region, followed by green MPs. Blue, red, transparent (Fig 9). The comparison of MP color in different sexes of *C. rhabdodactylus* collected from the intertidal region revealed that maximum MPs were black-colored in male crabs, followed by red, green, transparent, and others. In females, black-colored MPs were dominant, followed by red, green, orange, transparent, and others. In the subtidal region, the maximum percentage of MPs in males was black, followed by blue, green, red, and transparent. In females, black-colored MPs were dominant, followed by green, blue, red, transparent, and others (Fig 9). Similarly, black, blue, and red MPs were found predominantly in the sediment and water samples and collected from the intertidal and subtidal regions (Fig 10A and B).

#### 3.2.4 Polymer composition of MPs.

**Fig 11:**
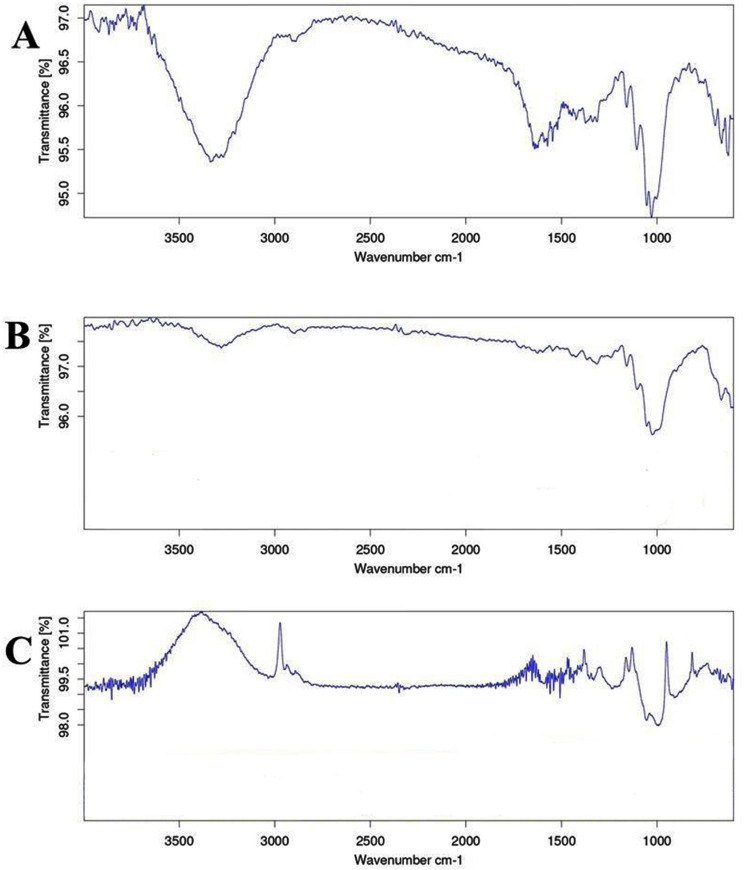
ATR-FTIR spectra for the obtained polymer: A) Polypropylene; B) Polyethylene; C) ethylene-vinyl acetate.

The spectra acquired from ATR-FTIR were compared to known plastic libraries, indicating that the majority of MPs were composed of polypropylene (PP) (50%), followed by polyethylene (PE) (30%) and ethylene-vinyl acetate (EVA) (20%) (Fig 11).

## 4. Discussion

MPs have been found worldwide, from glaciers in the polar region [39] to lakes [40], and from the atmosphere [41] to deep benthic habitats of the ocean [42]. Therefore, the presence of MPs in marine habitats is not surprising. Varying levels of MP contamination have been found in different groups of marine organisms, including plankton, worms, fish, crustaceans, mammals, and birds [16,30,31,43–46], depending on their mode of feeding and habitat. Therefore, the present study focuses on the variation in MP contamination levels in *C. rhabdodactylus* collected from the intertidal and subtidal habitats of the Gujarat coast, the first such study in India.

The present study found that the intertidal zone had significantly higher MP contamination, with a mean abundance of 11.16 ± 1.06 items/g; 6.44 items/individual, as compared to the subtidal zone (33 ± 0.34 items/g; 4.75 items/individual). Variation in the contamination rate of MPs between study sites may be due to the different levels of anthropogenic activities [47]. Therefore, MPs contamination can be found more prevalent in intertidal crabs than in subtidal crabs. Studies suggest that MPs are actively transported and accumulate primarily in the intertidal zone [48]. This further consolidation may be part of the food web of benthic organisms owing to biofouling processes [49], absorption into sea snow [50], and fecal matter [43]. Consequently, it causes increased MP contamination in benthic organisms, including the hermit crab species *C. rhabdodactylus*. However, subtidal sediments act as a potential sink for MPs [51], which have a relatively low abundance of MPs, reducing the contamination of MPs compared to the biota living in the intertidal zone.

In this investigation, MPs contamination was found to be significantly higher in the intertidal coastal environment of Gujarat, mostly due to the anthropogenic activities such as coastal tourism, fishing, and industrial discharge [28]. The intertidal zone acts as a natural deposition place for MPs, where tidal currents and wave action concentrate these particles in the sediments and water. Crabs in this region are particularly vulnerable because they can absorb microplastics (MPs) from the water when they respire. These particles can accumulate in their gills and intestines, as they often ingest sediment while feeding. This process of bioaccumulation begins with contamination of sediment and water ingestion from the environment, which allows the particles to first accumulate in the gastrointestinal tract and then in other tissues [52]. The subtidal regions are deeper and less directly affected by human activity, resulting in low levels of MP contamination in these areas and reduced ingestion by organisms. This highlights how habitat and human activities significantly influence the exposure and accumulation of MPs in marine organisms.

The calculated pollution indices indicate a higher level of microplastic contamination in the intertidal region compared to the subtidal zone. The CF highlights considerable contamination in intertidal areas, suggesting greater polymer accumulation. Both zones fall under risk category III based on the H index, indicating potential ecological threats. However, the PRI suggests the intertidal zone poses a very high risk, while the subtidal area reflects a medium risk, underscoring the need for focused mitigation in intertidal habitats.

The abundance of MPs contamination in *C. rhabdodactylus* was higher compared to other crab species, such as *P. sanguinolentus* (0.67 ± 0.62 items/g) [30], *Metacarcinus magister* (0.24 items/g) [53], and *Emerita analoga* (0.65 items/individual) [54]. The degree of contamination of MPs can fluctuate based on the various factors, including the size and age of the individual, the structure of the gastrointestinal tract, their feeding habit and methods [55,56]. Hermit crabs usually prefer a filter and deposit strategy feeding mechanism, making them susceptible to the unknown ingestion of MPs by mistake for food, which can then enter their gastrointestinal tract [11,12,57]. This feeding behaviour is likely to contribute significantly to the contamination rates of MPs found in these filter-feeding hermit crab species.

MPs contamination in female *C. rhabdodactylus*, both intertidal and subtidal regions, has been found to be significantly higher than that of male crabs. Similar results have been reported for *Streptocephalus proboscideus* [58]. Some other factors, including the availability of prey, energy demand, and seasonal variations, may affect the feeding habits of female crabs. Because women invest more energy in producing large gametes and incubating their eggs, they need to consume more food than males, leading to increased MP ingestion [59].

The MPs’ classification on shape revealed that most of the fibers were recorded from both intertidal (81.99%) and subtidal (55.38%) regions, followed by fragments (17.85% and 23.78%), and films (0.16% and 3.13%). In various other species, including *Carcinus aestuarii* [60], *Leptuca festae* [61], *Ocypode quadrata* [62], *Metopograpsus quadridentatus* [63], and *Pachygrapsus transversus* [64], the fibers abundance has been reported to be maximum. Fibers are the most abundant form of MPs particles found in the marine environment [21], which increases the possibility of microfibers being ingested by marine organisms [30]. The primary source of microfiber is often linked to fishing gears, such as nets and rope, as well as laundry and wastewater discharge from textile industries [65–68]. In addition, land and sea-based activities in coastal areas contribute to the accumulation of MPs in various shapes [48].

The size-wise classification of MPs found in *C. rhabdodactylus* indicated that the highest abundance was from smaller-sized classes. A similar conclusion has been reported in the study of various crab species, including *Chionoecetes opilio* [69], *Carcinus aestuarii* [59], *P. sanguinolentus* [30], *Mutata victor*, *Scylla serrata*, *Dotilla blanfordi*, and *Charybdis helleri* [59]. In addition, studies on prawns such as *Metapenaeus dobsoni, Penaeus indicus*, and *Solenocera crassicornis* have yielded comparable results [31]. Small-sized MPs are often mistaken for plankton, which increases the probability of their accidental ingestion and subsequent bioaccumulation by filter-feeding organisms [70,71], indicating the presence of MPs of different sizes that have fragmented into small particles with various shapes and sizes [72].

Black, blue, and green-colored MPs were observed to be dominantly recorded from the *C. rhabdodactylus* crabs in both coastal regions. Similar results have been observed in other studies, such as *Ocypode quadrata* [62], *Portunus pelagicus* [73], *Alepisaurus ferox* [74], *Neohelice granulata* [53], *Portunus trituberculatus*, *Charybdis japonica, Dorippe japonica*, and *Matuta planipes* [54]. Fishing gears, especially in blue and black colours used by fishermen, can act as a significant source of coloured MPs in the marine environment. Blue and black coloured MPs can easily camouflage with sea water and benthic substrata, making marine organisms more likely to ingest these coloured MPs [21,55]. The similarities in coloured can deceive marine fauna, which mistake them for their common prey, which increases the risk of ingestion during the hunting behaviour of MPs [75].

Different polymers are used in different types of plastic production to achieve specific colors and characteristics by including additives and pigments [76]. FTIR analysis shows that most MPs contain polypropylene (PP) and polyethylene (PE) polymer compounds, including ethylene-vinyl acetate (EVA) polymer. The identification of the polymer composition of the MPs is crucial to trace their roots in the marine environments. Potential sources of well-known plastic polymers in this area include food packets and packaging materials improperly disposed of by visitors. As well as abandoned fishing nets. Additionally, products such as medical devices, bags, fishing gear, clothing, marine appliances, and various packaging materials may contain polypropylene (PP) [77,78]. Possible sources of polyethylene (PE) in marine accommodation include fishing devices and wastewater from industries involved in the production of textiles [79].

Polyethylene may also be used to manufacture agricultural mulches, packaging films, shopping bags, squeeze bottles, wire-insulator materials, household items, toys, and other products [80]. EVA MPs can be formed by the disintegration of larger plastic waste, entering aquatic environments from cosmetics, clothing, and industrial manufacturing processes. The presence of MPs in coastal sediments and water indicates their presence in water bodies due to inflow from tributaries, urban runoff, recreational activities, and wastewater effluents from industries and households [81]. Understanding these diverse sources is crucial for effective management strategies to mitigate the environmental impacts of MPs.

## Conclusion

This study compared MP contamination in Gujarat intertidal and subtidal hermit crabs, *C. rhabdodactylus*. Comparative analysis revealed that intertidal crabs had a higher MP contamination rate than subtidal crabs. The level of contamination may vary due to MP accumulation in the intertidal and subtidal habitats. Male and female MP contamination levels differed significantly, likely because of different feeding rates for different energy needs. Physical analysis of MPs showed shape, size, and color variation. Chemical analysis of recovered MPs indicated that the major polymers were polypropylene (PP), polyethylene (PE), and ethylene-vinyl acetate (EVA). Toys, shopping bags, kitchenware, packing films, fishing gear, and other equipment are all potential sources of identifiable polymers in marine environments. This study recommends immediate plastic debris management in the marine ecosystem of Gujarat state. The results showed that this type of pollution can harm benthic invertebrates, especially intertidal and subtidal invertebrates. This study noted that MPs in hermit crabs can biomagnify and accumulate in predators and humans. This study emphasizes the accumulation in organisms at higher trophic levels, including predators and humans, due to biomagnification.

## Supporting information

S1 Table Pollution indices engaged in the study. (DOC)

S2 File Minimal data. Minimal data set. (XLSX)

## References

[1] Geyer R, Jambeck JR, Law KL. Production, use, and fate of all plastics ever made. Science Advances. 2017;3(7). 10.1126/sciadv.1700782 PMC5517107 28776036

[2] Europe P. Plastics—The Fast Facts. 2023.

[3] Lippiatt S, Opfer S, Arthur C. Marine debris monitoring and assessment: recommendations for monitoring debris trends in the marine environment. 2013.

[4] Kawsar MdA, Munny FJ, Saif UM, Harun-Al-Rashid A, Rahman MdA, Barman SK, et al. Unveiling the microplastic crisis: Insights into Bangladesh’s aquatic ecosystems - origins, impact, and solutions. J Hazardous Materials Advances. 2024;14:100430. doi: 10.1016/j.hazadv.2024.100430

[5] Yi YZ, Azman S, Primus A, Said MIM, Abideen MZ. Microplastic ingestion by crabs. In: The 6th proceeding of Civil Engineering School of Civil Engineering, 2021. 363–75.

[6] Zazouli M, Nejati H, Hashempour Y, Dehbandi R, Nam VT, Fakhri Y. Occurrence of microplastics (MPs) in the gastrointestinal tract of fishes: A global systematic review and meta-analysis and meta-regression. Sci Total Environ. 2022;815:152743. doi: 10.1016/j.scitotenv.2021.152743 35007572

[7] Lozano YM, Lehnert T, Linck LT, Lehmann A, Rillig MC. Microplastic shape, polymer type, and concentration affect soil properties and plant biomass. Front Plant Sci. 2021;12. 10.3389/fpls.2021.616645 PMC7920964 33664758

[8] Bayar J, Hashmi MZ, Khan MA, Pongpiachan S, Su X, Chakaraborty P. Emerging issue of microplastic in sediments and surface water in South Asia: A review of status, research needs, and data gaps. 2022. p. 3–19.

[9] Shinde R, Rabari V, Duggal R, Patel A, Alharbi SA, Ansari MJ. An assessment of microplastic contamination in beach sediment of Maharashtra State, India, with special reference to anthropogenic activities. Water Environment Research. 2024;96(5). 10.1002/wer.11033 38720414

[10] Yogi K, Rabari V, Patel K, Patel H, Trivedi J, Rakib MRJ. Gujarat’s plastic plight: unveiling characterization, abundance, and pollution index of beachside plastic pollution. Discover Oceans. 2024;1(1):8.

[11] Daniel DB, Ashraf PM, Thomas SN. Microplastics in the edible and inedible tissues of pelagic fishes sold for human consumption in Kerala, India. Environ Pollut. 2020;266(Pt 2):115365. doi: 10.1016/j.envpol.2020.115365 32814179

[12] Daniel DB, Ashraf PM, Thomas SN. Abundance, characteristics and seasonal variation of microplastics in Indian white shrimps (Fenneropenaeus indicus) from coastal waters off Cochin, Kerala, India. Sci Total Environ. 2020;737:139839. doi: 10.1016/j.scitotenv.2020.139839 32526586

[13] Hossain MS, Rahman MS, Uddin MN, Sharifuzzaman SM, Chowdhury SR, Sarker S, et al. Microplastic contamination in Penaeid shrimp from the Northern Bay of Bengal. Chemosphere. 2020;238:124688. doi: 10.1016/j.chemosphere.2019.124688 31524623

[14] Dowarah K, Patchaiyappan A, Thirunavukkarasu C, Jayakumar S, Devipriya SP. Quantification of microplastics using Nile Red in two bivalve species Perna viridis and Meretrix meretrix from three estuaries in Pondicherry, India and microplastic uptake by local communities through bivalve diet. Mar Pollut Bull. 2020;153:110982. doi: 10.1016/j.marpolbul.2020.110982 32275539

[15] Patterson J, Jeyasanta KI, Laju RL, Edward JKP. Microplastic contamination in Indian edible mussels (Perna perna and Perna viridis) and their environs. Mar Pollut Bull. 2021;171:112678. doi: 10.1016/j.marpolbul.2021.112678 34242958

[16] Rabari V, Patel H, Patel K, Patel A, Bagtharia S, Trivedi J. Quantitative assessment of microplastic contamination in muddy shores of Gulf of Khambhat, India. Mar Pollut Bull. 2023;192:115131. doi: 10.1016/j.marpolbul.2023.115131 37290300

[17] Doshi M, Rabari V, Patel A, Yadav VK, Sahoo DK, Trivedi J. A systematic review on microplastic contamination in marine Crustacea and Mollusca of Asia: Current scenario, concentration, characterization, polymeric risk assessment, and future prospectives. Water Environment Research. 2024;96(5). 10.1002/wer.11029 38708452

[18] Oza J, Rabari V, Yadav VK, Sahoo DK, Patel A, Trivedi J. A systematic review on microplastic contamination in fishes of Asia: polymeric risk assessment and future prospectives. Environmental Toxicology and Chemistry. 2024;43(4):671–85. 38353354 10.1002/etc.5821

[19] Joshi K, Rabari V, Patel H, Patel K, Rakib MRJ, Trivedi J, et al. Microplastic contamination in filter-feeding oyster Saccostrea cuccullata: Novel insights in a marine ecosystem. Mar Pollut Bull. 2024;202:116326. doi: 10.1016/j.marpolbul.2024.116326 38583217

[20] Osman AI, Hosny M, Eltaweil AS, Omar S, Elgarahy AM, Farghali M, et al. Microplastic sources, formation, toxicity and remediation: a review. Environ Chem Lett. 2023;21(4):1–41. doi: 10.1007/s10311-023-01593-3 37362012 PMC10072287

[21] Wright SL, Thompson RC, Galloway TS. The physical impacts of microplastics on marine organisms: a review. Environ Pollut. 2013;178:483–92. doi: 10.1016/j.envpol.2013.02.031 23545014

[22] Cunningham EM, Mundye A, Kregting L, Dick JTA, Crump A, Riddell G. Animal contests and microplastics: evidence of disrupted behaviour in hermit crabs Pagurus bernhardus. Royal Society Open Science. 2021;8(10). 10.1098/rsos.211089 PMC8511743 34659782

[23] Crump A, Mullens C, Bethell EJ, Cunningham EM, Arnott G. Microplastics disrupt hermit crab shell selection. Biol Lett. 2020;16(4):20200030. doi: 10.1098/rsbl.2020.0030 32343937 PMC7211466

[24] Nanninga GB, Horswill C, Lane SM, Manica A, Briffa M. Microplastic exposure increases predictability of predator avoidance strategies in hermit crabs. J Hazardous Materials Letters. 2020;1:100005. doi: 10.1016/j.hazl.2020.100005

[25] Patel KJ, Vachhrajani KD, Trivedi JN. Population structure and reproductive biology of Clibanarius rhabdodactylus Forest, 1953 (Crustacea: Anomura: Diogenidae) in Gujarat state, India. Regional Studies in Marine Science. 2023;63:103033. doi: 10.1016/j.rsma.2023.103033

[26] Saravanan KR, Sivakumar K, Choudhury BC. Important coastal and marine biodiversity areas of India. ENVIS Bull Wildl Protect Areas. 2013;15:134–88.

[27] Patel K, Padate V, Osawa M, Tiwari S, Vachhrajani K, Trivedi J. An annotated checklist of anomuran species (Crustacea: Decapoda) of India. Zootaxa. 2022;5157(1):1–100. doi: 10.11646/zootaxa.5157.1.1 36095562

[28] Rabari V, Patel K, Patel H, Trivedi J. Quantitative assessment of microplastic in sandy beaches of Gujarat state, India. Mar Pollut Bull. 2022;181:113925. doi: 10.1016/j.marpolbul.2022.113925 35841675

[29] Prusty K, Rabari V, Patel K, Ali D, Alarifi S, Yadav VK. An assessment of microplastic contamination in a commercially important marine fish, Harpadon nehereus (Hamilton, 1822). Fishes. 2023;8(9):432.

[30] Rabari V, Patel H, Ali D, Yadav VK, Patel A, Sahoo DK. Ingestion and polymeric risk assessment of microplastic contamination in commercially important brachyuran crab Portunus sanguinolentus. Front Marine Sci. 2023;10.

[31] Rabari V, Rakib MRJ, Patel H, Idris AM, Malafaia G, Trivedi J. Microplastic prevalence in epipelagic layer: Evidence from epipelagic inhabiting prawns of north-west Arabian Sea. Mar Pollut Bull. 2024;200:116137. doi: 10.1016/j.marpolbul.2024.116137 38377866

[32] Patel P, Patel K, Trivedi J. First record of Hermit crab Clibanarius ransoni Forest, 1953 (Crustacea: Anomura: Diogenidae) from India. J Biol Study. 2020;3(1):19–23. doi: 10.62400/jbs.v3i1.4601

[33] Hazlett BA. Stimuli involved in the feeding behavior of the hermit crab clibanarius vittatus (Decapoda, Paguridea). Crustac. 1968;15(3):305–11. doi: 10.1163/156854068x00430

[34] Das S, Chatterjee NH, Choudhury A, Ray A, Rana N, Banerjee A, et al. Characterization and ecological risk assessment of microplastics accumulated in sea water, sand, sediment, shell water and selected tissues of hermit crab of Sundarban Biosphere Reserve. Environ Pollut. 2024;357:124484. doi: 10.1016/j.envpol.2024.124484 38960120

[35] Thacker D, Patel KJ, Patel PR, Trivedi JN. Gastropod shell occupation pattern of hermit crab Clibanarius rhabdodactylus Forest, 1953 in the infralittoral zone of Gulf of Kachchh, Gujarat, India. Uttar Pradesh J Zoology. 2021;42(5):20–31.

[36] Kachhiya P, Raval J, Poriya P, Kundu R. Diversity and new records of intertidal hermit crabs of the genus Clibanarius (Crustacea: Decapoda: Diogenidae) from Gujarat coast off the northern Arabian Sea, with two new records for the mainland Indian coastline. J Threat Taxa. 2017;9(6):10334. doi: 10.11609/jott.2268.9.6.10334-10339

[37] Hara J, Frias J, Nash R. Quantification of microplastic ingestion by the decapod crustacean Nephrops norvegicus from Irish waters. Mar Pollut Bull. 2020;152:110905. doi: 10.1016/j.marpolbul.2020.110905 31957681

[38] Xu S, Ma J, Ji R, Pan K, Miao A-J. Microplastics in aquatic environments: Occurrence, accumulation, and biological effects. Sci Total Environ. 2020;703:134699. doi: 10.1016/j.scitotenv.2019.134699 31726297

[39] Ambrosini R, Azzoni RS, Pittino F, Diolaiuti G, Franzetti A, Parolini M. First evidence of microplastic contamination in the supraglacial debris of an alpine glacier. Environ Pollut. 2019;253:297–301. doi: 10.1016/j.envpol.2019.07.005 31323612

[40] Eriksen M, Mason S, Wilson S, Box C, Zellers A, Edwards W, et al. Microplastic pollution in the surface waters of the Laurentian Great Lakes. Mar Pollut Bull. 2013;77(1–2):177–82. doi: 10.1016/j.marpolbul.2013.10.007 24449922

[41] Gasperi J, Wright SL, Dris R, Collard F, Mandin C, Guerrouache M, et al. Microplastics in air: Are we breathing it in?. Curr Opin Environ Sci Health. 2018;1:1–5. doi: 10.1016/j.coesh.2017.10.002

[42] Wang R, Zhang C, Huang X, Zhao L, Yang S, Struck U, et al. Distribution and source of heavy metals in the sediments of the coastal East China sea: Geochemical controls and typhoon impact. Environ Pollut. 2020;260:113936. doi: 10.1016/j.envpol.2020.113936 32041006

[43] Cole M, Lindeque PK, Fileman E, Clark J, Lewis C, Halsband C. Microplastics alter the properties and sinking rates of zooplankton faecal pellets. Environ Sci Technol. 2016;50(6):3239–46. 26905979 10.1021/acs.est.5b05905

[44] Ferreira P, Fonte E, Soares ME, Carvalho F, Guilhermino L. Effects of multi-stressors on juveniles of the marine fish Pomatoschistus microps: Gold nanoparticles, microplastics and temperature. Aquat Toxicol. 2016;170:89–103. doi: 10.1016/j.aquatox.2015.11.011 26642093

[45] Youngren SM, Rapp DC, Hyrenbach KD. Plastic ingestion by Tristram’s Storm-petrel (Oceanodroma tristrami) chicks from French frigate shoals, Northwestern Hawaiian Islands. Mar Pollut Bull. 2018;128:369–78. doi: 10.1016/j.marpolbul.2018.01.053 29571385

[46] Nelms SE, Barnett J, Brownlow A, Davison NJ, Deaville R, Galloway TS. Microplastics in marine mammals stranded around the British coast: ubiquitous but transitory?. Sci Rep. 2019;9(1):1075. 30705316 10.1038/s41598-018-37428-3 PMC6355900

[47] Horn D, Miller M, Anderson S, Steele C. Microplastics are ubiquitous on California beaches and enter the coastal food web through consumption by Pacific mole crabs. Mar Pollut Bull. 2019;139:231–7. doi: 10.1016/j.marpolbul.2018.12.039 30686424

[48] Browne MA, Galloway TS, Thompson RC. Spatial patterns of plastic debris along estuarine shorelines. Environ Sci Technol. 2010;44(9). 10.1021/es903784e 20377170

[49] Andrady AL. Microplastics in the marine environment. Mar Pollut Bull. 2011;62(8):1596–605. doi: 10.1016/j.marpolbul.2011.05.030 21742351

[50] Woodall LC, Sanchez-Vidal A, Canals M, Paterson GLJ, Coppock R, Sleight V, et al. The deep sea is a major sink for microplastic debris. R Soc Open Sci. 2014;1(4):140317. doi: 10.1098/rsos.140317 26064573 PMC4448771

[51] Pagter E, Frias J, Kavanagh F, Nash R. Varying levels of microplastics in benthic sediments within a shallow coastal embayment. Estuarine, Coastal and Shelf Science. 2020;243:106915. doi: 10.1016/j.ecss.2020.106915

[52] Zala H, Rabari V, Patel K, Patel H, Yadav VK, Patel A, et al. Microplastic from beach sediment to tissue: a case study on burrowing crab Dotilla blanfordi. PeerJ. 2024;12:e17738. doi: 10.7717/peerj.17738 39011379 PMC11249004

[53] Villagran DM, Truchet DM, Buzzi NS, Forero Lopez AD, Fernández Severini MD. A baseline study of microplastics in the burrowing crab (Neohelice granulata) from a temperate southwestern Atlantic estuary. Mar Pollut Bull. 2020;150:110686. doi: 10.1016/j.marpolbul.2019.110686 31744606

[54] Zhang T, Sun Y, Song K, Du W, Huang W, Gu Z, et al. Microplastics in different tissues of wild crabs at three important fishing grounds in China. Chemosphere. 2021;271:129479. doi: 10.1016/j.chemosphere.2020.129479 33460905

[55] Rezania S, Park J, Md Din MF, Mat Taib S, Talaiekhozani A, Kumar Yadav K, et al. Microplastics pollution in different aquatic environments and biota: A review of recent studies. Mar Pollut Bull. 2018;133:191–208. doi: 10.1016/j.marpolbul.2018.05.022 30041307

[56] Abbasi S, Soltani N, Keshavarzi B, Moore F, Turner A, Hassanaghaei M. Microplastics in different tissues of fish and prawn from the Musa Estuary, Persian Gulf. Chemosphere. 2018;205:80–7. doi: 10.1016/j.chemosphere.2018.04.076 29684694

[57] Ningrum EW, Patria MP. Ingestion of microplastics by anchovies from east Lombok Harbour, Lombok Island, Indonesia. 2019. 040002.

[58] Jawahar Ali A, Sarma SSS, Murugan G, Dumont HJ. Effect of zooplankton type and abundance on prey consumption by the fairy shrimp, Streptocephalus proboscideus (Anostraca: Crustacea). Hydrobiologia. 1996;319(3):191–202. doi: 10.1007/bf00013732

[59] Patel M, Rabari V, Acharya CA, Trivedi JN. A Comparative study on microplastic contamination in four brachyuran crabs along the Bhavnagar Coast. In: Proceedings of National Seminar on Emerging Trends in Life Sciences, 2024. 203–20.

[60] Piarulli S, Scapinello S, Comandini P, Magnusson K, Granberg M, Wong JXW, et al. Microplastic in wild populations of the omnivorous crab Carcinus aestuarii: A review and a regional-scale test of extraction methods, including microfibres. Environ Pollut. 2019;251:117–27. doi: 10.1016/j.envpol.2019.04.092 31075692

[61] Villegas L, Cabrera M, Capparelli MV. Assessment of microplastic and organophosphate pesticides contamination in fiddler crabs from a ramsar site in the estuary of guayas river, Ecuador. Bull Environ Contam Toxicol. 2021;107(1):20–8. doi: 10.1007/s00128-021-03238-z 33891142

[62] Costa LL, Arueira VF, da Costa MF, Di Beneditto APM, Zalmon IR. Can the Atlantic ghost crab be a potential biomonitor of microplastic pollution of sandy beaches sediment?. Mar Pollut Bull. 2019;145:5–13. doi: 10.1016/j.marpolbul.2019.05.019 31590817

[63] Patria MP, Santoso CA, Tsabita N. Microplastic ingestion by periwinkle snail Littoraria scabra and mangrove crab Metopograpsus quadridentata in Pramuka Island, Jakarta Bay, Indonesia. Sains Malays. 2020;49(09):2151–8.

[64] Matheus SF de B, Tereza C dos SC, Alberis SS, Ewerton V dos S. Ingestion of plastic debris affects feeding intensity in the rocky shore crab Pachygrapsus transversus Gibbes 1850 (Brachyura: Grapsidae). Int J Biodiversity Conserv. 2020;12(2):113–7.

[65] Güven O, Gökdağ K, Jovanović B, Kıdeyş AE. Microplastic litter composition of the Turkish territorial waters of the Mediterranean Sea, and its occurrence in the gastrointestinal tract of fish. Environ Pollut. 2017;223:286–94. doi: 10.1016/j.envpol.2017.01.025 28117186

[66] Feng Z, Zhang T, Li Y, He X, Wang R, Xu J, et al. The accumulation of microplastics in fish from an important fish farm and mariculture area, Haizhou Bay, China. Sci Total Environ. 2019;696:133948. doi: 10.1016/j.scitotenv.2019.133948 31442723

[67] Pedà C, Romeo T, Panti C, Caliani I, Casini S, Marsili L, et al. Integrated biomarker responses in European seabass Dicentrarchus labrax (Linnaeus, 1758) chronically exposed to PVC microplastics. J Hazard Mater. 2022;438:129488. doi: 10.1016/j.jhazmat.2022.129488 35999717

[68] Welden NA, Abylkhani B, Howarth LM. The effects of trophic transfer and environmental factors on microplastic uptake by plaice, Pleuronectes plastessa, and spider crab, Maja squinado. Environ Pollut. 2018;239:351–8. doi: 10.1016/j.envpol.2018.03.110 29674213

[69] Fang C, Zheng R, Zhang Y, Hong F, Mu J, Chen M, et al. Microplastic contamination in benthic organisms from the Arctic and sub-Arctic regions. Chemosphere. 2018;209:298–306. doi: 10.1016/j.chemosphere.2018.06.101 29933166

[70] Ory NC, Gallardo C, Lenz M, Thiel M. Capture, swallowing, and egestion of microplastics by a planktivorous juvenile fish. Environ Pollut. 2018;240:566–73. doi: 10.1016/j.envpol.2018.04.093 29758531

[71] Naidoo T, Sershen, Thompson RC, Rajkaran A. Quantification and characterisation of microplastics ingested by selected juvenile fish species associated with mangroves in KwaZulu-Natal, South Africa. Environ Pollut. 2020;257:113635. doi: 10.1016/j.envpol.2019.113635 31767237

[72] Sathish N, Jeyasanta KI, Patterson J. Abundance, characteristics and surface degradation features of microplastics in beach sediments of five coastal areas in Tamil Nadu, India. Mar Pollut Bull. 2019;142:112–8. doi: 10.1016/j.marpolbul.2019.03.037 31232283

[73] Kleawkla N. Microplastic fragments in stomach content of blue swimming crab, portunus pelagicus from Wonnapha Coastal Wetland, Chonburi Province, Thailand. Ramkhamhaeng Int J Sci Technol. 2019;2(3):7–16.

[74] Gago J, Portela S, Filgueiras AV, Salinas MP, Macías D. Ingestion of plastic debris (macro and micro) by longnose lancetfish (Alepisaurus ferox) in the North Atlantic Ocean. Regional Studies in Marine Science. 2020;33:100977. doi: 10.1016/j.rsma.2019.100977

[75] Kain EC, Lavers JL, Berg CJ, Raine AF, Bond AL. Plastic ingestion by Newell’s (Puffinus newelli) and wedge-tailed shearwaters (Ardenna pacifica) in Hawaii. Environ Sci Pollu Res. 2016;23(23):23951–8. 10.1007/s11356-016-7613-1 27638797

[76] Al-Malaika S, Axtell F, Rothon R, Gilbert M. Additives for plastics. Brydson’s plastics materials. Elsevier. 2017. p. 127–68.

[77] Maddah HA. Polypropylene as a promising plastic: A review. Am J Polymer Sci. 2016;6(1):1–11.

[78] Jung J-W, Park J-W, Eo S, Choi J, Song YK, Cho Y, et al. Ecological risk assessment of microplastics in coastal, shelf, and deep sea waters with a consideration of environmentally relevant size and shape. Environ Pollut. 2021;270:116217. doi: 10.1016/j.envpol.2020.116217 33359873

[79] Wang W, Gao H, Jin S, Li R, Na G. The ecotoxicological effects of microplastics on aquatic food web, from primary producer to human: A review. Ecotoxicol Environ Saf. 2019;173:110–7. doi: 10.1016/j.ecoenv.2019.01.113 30771654

[80] Ronca S. Polyethylene. In: Brydson’s Plastics Materials. Elsevier; 2017. p. 247–78.

[81] Hollerová A, Hodkovicová N, Blahová J, Faldyna M, Maršálek P, Svobodová Z. Microplastics as a potential risk for aquatic environment organisms – a review. Acta Vet Brno. 2021;90(1):99–107. doi: 10.2754/avb202190010099

