# Expression of Concern: A comparative analysis of microplastic contamination in hermit crab *Clibanarius rhabdodactylus* Forest, 1953, inhabiting intertidal and subtidal Coastal habitat of Gujarat state

**DOI:** 10.1371/journal.pone.0354209

**Published:** 2026-07-21

**Authors:** The PLOS One Editors

After this article [1] was published, concerns were noted that the articles cited as References 13 and 30 were retracted after [1] was published.

In addition, the *PLOS One* Editors have concerns about the article’s compliance with the PLOS Authorship policy.

In light of the cumulative issues, the *PLOS One* Editors issue this Expression of Concern. Readers are advised to interpret the article [1] with caution.
