# Supplementary material for: A comparative analysis of microplastic contamination in hermit crab Clibanarius rhabdodactylus Forest, 1953, inhabiting intertidal and subtidal Coastal habitat of Gujarat state
Source: PLoS One. 2025 Jun 12;20(6):e0325324. doi: 10.1371/journal.pone.0325324 (PMC12161533; doi:10.1371/journal.pone.0325324)
Supplement: S1 Table — (DOC) [file pone.0325324.s001.doc]

Table 1: Pollution indices engaged in the study.

| **Contamination factor (CF)** | **Risk category** | **Polymeric risk assessment (H)** | **Risk category** | **PRI** | **Risk category** |
| --- | --- | --- | --- | --- | --- |
| <1 | Low contamination | <10 | I | <150 | Low |
| 1–3 | Moderately contamination | 10–100 | II | 150–300 | Medium |
| 3–6 | Considerably contamination | 101–1000 | III | 300–600 | Considerable |
| ≥6 | Very highly contamination | 1001–10,000 | IV | 600–1200 | High |
|  |  | >10,000 | V | >1200 | Very high |
